# Applications of Silver Nanoparticles in Dentistry

**DOI:** 10.7759/cureus.44090

**Published:** 2023-08-25

**Authors:** Akarsh Bolenwar, Amit Reche, Nutan Dhamdhere, Samruddhi Rathi

**Affiliations:** 1 Public Health Dentistry, Sharad Pawar Dental College and Hospital, Datta Meghe Institute of Higher education and Research, Wardha, IND

**Keywords:** silver nanoparticles, agnps toxicity, endodontics, dental prosthesis, dental implantology, nanocomposites

## Abstract

Silver nanoparticles (AgNPs) have been effectively applicable in most regions because of their antimicrobial activity against various microorganisms. AgNPs can be applied in disinfection and prophylaxis and to prevent diseases of the oral cavity. Because of developing interest in AgNPs, this article shows the application of AgNPs in various fields of dentistry such as nanocomposites, implant coatings, inhibiting the growth of bacteria, preventing dental caries, biofilm, infectious microbes, local anesthesia, microorganisms causing damage to the pulp, as well as deals with diseases such as oral malignancies due to their antitumor properties. AgNPs have a potential system with major features including antibacterial, anti-inflammatory, and anticancer action. They may also be used as a sustained drug delivery vehicle.

## Introduction and background

Silver has been used in dental treatment for ages and became widely accepted in the mid-19th century as one of the primary ingredients in dental filling material used for tooth rehabilitation. Since 1930, aesthetic polymer-based resins gradually replaced them, and their use in amalgams has decreased [1]. Since nanoscience has advanced, outstanding antibacterial qualities of silver-based formulations with nanostructures have been effective against pathogens such as bacteria, viruses, and fungi. There is a resurgence in interest in silver, and some exciting new technologies, particularly in dental materials, are now being developed [2]. Silver nanoparticles (AgNPs) have a tendency to inhibit the growth of bacteria in prosthetics, materials, and implants to inhibit biofilm development, enhance caries arrestment, and induce osteogenic tissue [3-4]. The release of monovalent cation in silver and its oxidizing potential is primarily involved in how AgNPs work. Its synthesis and mode of action can both be impacted by particle size and shape. The effectiveness of AgNP antagonists toward a number of drug-resistant pathogens has previously been established. It would be directly applied with the intention of cleaning and protecting against harmful bacteria in the oral cavity [5]. These particles' primary application is for preventive purposes. AgNPs with precise target functionalities, regulated size and shape, and great homogeneity are now feasible. Compared to other antimicrobial compounds, AgNPs have a very strong antibacterial action and good biocompatibility [6]. Additionally, they can work in combination with certain antibiotics. The perspectives on AgNPs-based mouth care technologies are shown in detail in this review and are categorized according to their use in various branches of dentistry. Silver nanoparticles have a wide range of applications and uses, some of which are use in nanocomposites and implant coatings, as well as for antibacterial efficacy against cariogenic pathogens. Some nanoparticles are believed to have antitumor activities and hence can be used for cancer treatment and prevention [7-8].

## Review

Search methodology

We conducted a review through PubMed and Google Scholar in July 2022 using keywords such as “silver-nanoparticles” (title/abstract), “Nanocomposites,” “Dental Implantology,” “Dental prosthesis,” and “AgNPs Toxicity.” We additionally searched for key references from bibliographies of the relevant studies. The search was updated in February 2023. The reviewer monitored the retrieved studies against the inclusion and exclusion criteria in the beginning based on title and abstract and then on full texts. For inclusion, both published and unpublished studies in the English language were considered. We excluded studies published in other languages because of resource limitations and full-text article were unavailable to the reviewer.

Silver nanoparticles

Silver has a tendency to accept an electron pair; therefore, it has a natural tendency to act with a donor component, such as biological and organic molecules of phosphorus and sulfur, which are important building blocks for proteins, DNA, and cell membranes [9]. As a result, AgNPs can build up on the polysaccharide lying outside the plasma membrane of the cells of bacteria and fungi, causing evident changes with cytoplasmic shrinkage and membrane expansion. During the killing process, AgNP adherence improves membrane rigidity, transitions it from an ordered to a disordered state, and decomposes membrane components such as fatty acids, proteins, and carbohydrates. AgNP has the capacity to bind to membrane proteins, such as those involved in the respiratory chain and transport proteins, which can significantly affect membrane permeability, and ion transport [10-11]. AgNP may also enter bacteria, where it interacts with components and macromolecules such as fats, polypeptides, and DNA to impede protein synthesis and, furthermore, disturb cell wall activity [12-13].

Similar to other silver-containing compounds, AgNPs’ biological activity is caused by the slow release of silver as a result of redox reduction [14]. Additionally, the dimensions and structure of nanoparticles have a role in the suppression of cell growth against bacteria, fungi, and viruses, with diameters less than 10 nm having the strongest antimicrobial effects [15]. Amorphous calcium AgNP-phosphate, Chitalac-Ag, AgNP-methyl polymethylmethacrylate, fluorides, and other composites are generally used in combination with AgNPs. It can also be used on its own as a silver plasma or AgNP [16-17].

Synthesis of Silver Nanoparticles

AgNPs are manufactured using a precursor (often silver nitrate), a reducing agent that converts silver ions from Ag+ to Ag0, because metallic nanoparticles have a high surface energy. As a result, AgNP manufacturing might be chemical, physical, or biological [18]. The manufacturing of AgNP is based on the chemical reduction of Ag+1 to Ag0. The reduction agents and buffers used, such as sodium citrate, ascorbate, elemental hydrogen, polyol process, Tollens reagent, and poly (ethylene glycol)-block copolymers, distinguish distinct chemical processes. Several protective agents (stabilizers) such as thiols, amines, acids, alcohols, and polymeric compounds such as chitosan and polymethylmethacrylate have been also used. UV irradiation, thermal synthesis, and spray pyrolysis are employed in physical synthesis [19-20].

Mechanisms of Action of AgNPs

Nanoparticles have the tendency to inhibit the growth of bacteria because of their expanded surface region, giving optimum contact with microorganisms when contrasted with other growth-inhibiting agents [21]. AgNP stimulates the production of an oxygen-containing unsteady molecule that readily interacts with other particles in a cell. The main species responsible for the effects of exposure to ionizing radiation and various chemical agents are hydroxyl radicals [5]. They suppress or inhibit the growth of various gram bacteria. The plasma membrane can break down and vary in permeability when these nanoparticles interact with membrane proteins and phospholipids [22].

Uses of AgNPs in various branches of dentistry

Silver ions have a range of purposes and are employed in dentistry because of their antimicrobial properties. Its action is primarily due to the gradual release of its ions. Studies have suggested the use of nanoparticles in a variety of branches of dentistry, including oral microbiology, prosthodontics, orthodontics, endodontics, periodontics, and preventive dentistry [23].

Oral Microbiology

More than 700 bacterial species can be found in the oral cavity. This microorganism plays a vital role in determining the etiology of various oral and systemic disorders. According to research, Streptococcus mutans, Staphylococcus aureus, Streptococcus sobrinus, Lactobacillus acidophilus, Lacticaseibacillus casei, Streptococcus sanguinis, Enterococcus faecalis, and Actinomyces actinomycetemcomitans are all resistant to AgNPs antibacterial effect [24]. Due to the multispecies biofilms that cause caries in the oral cavity, conducting tests and investigations on the therapeutic use of AgNPs is difficult. The antibacterial efficiency of nanosilver is proportional to the size of its particles. Smaller diameter AgNPs inhibit biofilms better than larger particles and are efficient against S. mutants and Streptococcus oralis biofilms [25].

Pedodontics and Preventive Dentistry

It is an age-specific branch that offers complete prevention and oral health care for newborn, children, and young individuals, as well as those who require special medical requirements. AgNPs have been shown to be effective antibacterial agents in the prevention of dental cavities in children. When compared to the usual sealant, the AgNP mixed with pit and fissure sealant greatly reduced tooth demineralization and presumably boosted remineralization. Glass ionomer cement (GIC) is used in pediatric and restorative dentistry, which is familiar for its capability to release and store fluoride. It also show anticaries properties. A biomaterial having antibacterial activity against both types of gram bacteria was produced via the interaction of GIC with AgNP [5]. AgNP-associated two-step adhesive systems surpassed self-etchers in terms of shear strength in terms of antibacterial activity of the self-etching adhesive. Commercial products that are compatible and less toxic to stem cells from dental pulp were produced as a result of AgNP integration in disinfectants [26]. Silver ions may penetrate and aggregate in carious lesions, causing enamel hardening. Dentists employ fluoride-based varnish in the therapeutic environment to remineralize incipient lesions. However, when 5% AgNPs are added to sodium fluoride varnish, the spread of caries lesions in residual teeth is inhibited by 77%, without the presence of a metallic taste or unpleasant ulcerations [27].

Orthodontics

The cleaning procedure is hampered by fixed orthodontic devices used on tooth surfaces, which results in the formation of dental biofilm. Plaque formation is caused by an increase in the number of microbacterial activity in saliva and the oral cavity following the use of equipment [28]. White spots and other carious lesions are the most prevalent problem with these kinds of devices. To avoid these kind of problems, AgNP was applied to elastomeric modules, brackets, orthodontic wire, and titanium microimplants. Additionally, acrylic resins and the base plates of orthodontic equipment were made with nanoparticles, which prevented the growth of planktonic organisms and biofilms. AgNPs and GIC composites used in orthodontic cementation also minimize the metabolic activity of the biofilm and the formation of bacterial acid [29].

Endodontics

Pathological pulp, which is a result of microbial colonization and may potentially induce bone infection, is the primary cause of apical periodontitis. A facultative anaerobic gram-positive bacteria frequently found in infected root canals causes chronic and persistent infections. Through the release of silver ions, the interaction of AgNP with composites inhibits gram-positive bacteria colonization [30]. As an irrigant, AgNPs were more biocompatible than 2.5% sodium hypochlorite (NaOCl) at low concentrations. AgNPs are effective against E. faecalis and can thus be employed as an alternative to NaOCl. AgNP has potential antibacterial activity against a range of gram-negative bacteria when combined with Ca-based cement and mineral trioxide aggregate (MTA). Silver particles can reduce microbial adherence to the tooth surface and improve the antibacterial action of endodontic sealers. Additionally, these particles enhanced the MTA radiopacity [31].

Restorative Dentistry

Secondary caries of the dental restoration may occur from remnant bacteria in the prepared tooth cavity and colonized bacteria in microleakages along tooth-restoration interfaces. Adhesive methods and resin composites can benefit from the use of AgNPs and may prevent secondary caries by exerting significant antibacterial effects at low concentrations. Due to the extensive inhibitory activity of cariogenic bacteria, restorative adhesives comprising AgNPs may interfere with biofilm development. Because the silver ions produced per day are relatively low, the composite resin with AgNPs exhibited no apparent negative effect on fibroblasts [32].

Periodontics

AgNPs can have an impact on various oral tissue and cell types in addition to the bacteria that cause tooth caries. Thus, some studies show the effects of nanoparticles on human oral keratinocytes and gingival fibroblasts. The key challenge discovered when investigating the use of AgNP in dentistry is establishing the appropriate concentration that is toxic to bacteria but not cytotoxic to the patient's cells, assuring that no harm to healthy tissues occurs. A significant study revealed that 2-nm AgNPs at 1.5 ug/mL have no cytotoxic action [33]. The interaction of AgNPs with fluorine creates catabolic pressure in gingival fibroblasts, causing inflammation of tissue and cells, which initiates apoptosis [34].

Prosthodontics

Essentially, it is split into two subfields: dental implantology and dental prosthetics.

Dental implantology: Peri-implantitis is an infectious disease that causes a loss of marginal bone around functioning implant, which leads to the development of bacterial biofilm, and is one of the most frequent reasons for implant failure. AgNP-based composites applied to titanium discs reduced microbial lactate generation and biofilm adherence. AgNP is effective against Escherichia coli when it is coated on titanium discs with hydroxyapatite, and the amount of AgNP is determined by the rate of transformation in the electrochemical circuit of metallic cation to metals by electron transfer [35]. AgNPs with an antibacterial activity and nontoxic nature toward human dental pulp stem cells may be produced directly on titanium plates. The tensile strength of a membrane coated with AgNPs was improved through directed tissue regeneration. AgNPs and natural rubber membrane together resulted in reduced cytotoxicity and 98% cell survival [36].

Dental prosthesis: Dentistry uses a variety of materials based on silicones, alginates, and porcelain to manufacture mold and prosthetic devices. When AgNPs are added to silicones, the antibacterial action increases without affecting the properties of impression material. The addition of AgNP to alginates reduced the setting time and improved cement solubilization. When AgNPs are added to an ethylene-vinyl acetate copolymer, they restrict the growth of different bacteria and produce a bacteriostatic effect [37]. Porcelain with AgNPs added to it has a higher fatigue parameter and greater fracture resistance. In geriatric patients, we use AgNPs in dental implants and denture base resins and as tissue conditioners [38].

Other Applications of AgNPs in Dentistry

In addition to the applications in dental uses already stated, AgNPs have also been researched as intriguing techniques for the treatment and detection of various malignancies. Squamous cell carcinoma (SSC) is one of the most frequently occurring malignancy that affects the oral cavity. This cancer makes up 90% of head and neck cancers and includes the class of epithelial malignancies that begin in the oral or nasal cavity, paranasal sinuses, lips, pharynx, or larynx [39]. AgNPs’ anticancer qualities in particular diseases are responsible for their application in the treatment of cancer. This characteristics of nanoparticles are linked with their ability to induce catabolic pressure, which leads to DNA damage and death of cells. It has been demonstrated that the anticancer drug quinacrine was more effective against oral cancer cells and oral SSC stem cells when it was combined with hybrid AgNPs [40]. This would assist in enhancing disease therapy and diagnosis, and, in turn, patients' quality of life [41].

Toxicity of AgNPs

AgNPs improve the effectiveness of restorative materials, but they can also be employed to create situation suitable for using AgNPs in dentistry. The activity of free Ag+ions released in the environment was shown to positively correspond with the toxicity of AgNPs [42]. The capacity of AgNPs to pass the blood-brain barrier and potentially accumulate in the brain is another area of concern. Studies conducted in vitro revealed that AgNPs might cause a decreased mitochondrial activity in a variety of cell types, including human skin cancer, hepatic cells, murine neuroblastoma cells, and epidermal keratinocytes, and it also has the impact of these AgNPs on inflammatory processes, thrombosis, and coronary artery diseases [43].

## Conclusions

The use of AgNPs in dentistry definitely has the potential to improve patient outcomes. It has outstanding antimicrobial activity when combined with materials such as nanocomposites, acrylic resins, resin co-monomers, adhesives, and implant coatings. Also, few clinical studies have investigated the therapeutic efﬁcacy of AgNPs and caries-preventing properties of these particles. The broad-spectrum antimicrobial properties of AgNPs have been well recognized and used in wound healing. Composites, and dental and orthodontic adhesives containing AgNPs are capable of inhibiting biofilm without altering their bond strength properties. By producing intercellular reactive oxygen species (ROS), damaging DNA, and stimulating signalling cascades in cells, AgNPs have the potential to cause cytotoxicity, genotoxicity, and an inflammatory response that eventually results in cell death.
